# The Spectrum of Genetic Causes of Familial Hypercholesterolemia Phenotype

**DOI:** 10.1007/s11883-026-01435-x

**Published:** 2026-07-04

**Authors:** M. Bourbon, A. C. Alves, J. R. Chora, A. J. Hooper, M. Abifadel

**Affiliations:** 1 Unidade de Investigação e Desenvolvimento, Grupo de Investigação Cardiovascular, Departamento de Promoção da Saúde e Prevenção de Doenças Não Transmissíveis, Instituto Nacional de Saúde Doutor Ricardo Jorge, Lisbon, Portugal; 2 Cardiovascular Centre of the University of Lisbon (CCUL), Faculty of Medicine, University of Lisbon, Lisbon, Portugal; 3 Department of Clinical Biochemistry, PathWest Laboratory Medicine WA, Royal Perth Hospital and Fiona Stanley Hospital Network, University of Western Australia, Perth, WA Australia; 4 Laboratory of Biochemistry and Molecular Therapeutics, Faculty of Pharmacy, Saint Joseph University of Beirut, Beirut, Lebanon; 5 Sorbonne Université, INSERM, Foundation for Innovation in Cardiometabolism and Nutrition (ICAN), UMR_S1166, Paris, France

**Keywords:** Genetic testing, Familial hypercholesterolemia, Next-generation sequencing, Phenotype – genotype, personalised medicine

## Abstract

**Purpose of Review:**

This paper reviews the genetic spectrum underlying the Familial Hypercholesterolemia (FH) phenotype, aiming to improve diagnosis, awareness and understanding of these inherited lipid disorders.

**Recent Findings:**

Although genetic testing for FH has traditionally targeted *LDLR*, *APOB*, and *PCSK9*, the adoption of an expanded eight-gene sequencing panel is now recommended. This broader approach improves diagnostic accuracy by identifying additional lipid disorders that mimic the FH phenotype. Among these, sitosterolemia and the *APOE* variant p.(Leu167del) appear to be more prevalent than previously recognized. *LDLR* variants remain the predominant cause of FH, yet the functional significance of approximately half of the reported variants remains undetermined. Recent research efforts are increasing and aimed at resolving these uncertainties through functional characterization studies. Penetrance varies markedly among FH genes, with *LDLR* variants showing highest penetrance (> 90%), *APOB* intermediate (~ 50–70%), and *PCSK9* is gain-of-function dependent, influencing phenotype severity.

**Summary:**

Familial hypercholesterolemia (FH) remains widely underdiagnosed and undertreated, increasing preventable cardiovascular risk. Advances in genetic testing with expanded multi-gene panels have enhanced diagnostic precision, but accurate variant classification—using ACMG guidelines and functional assays—is crucial to reduce variants of uncertain significance. Expanded FH genetic testing not only differentiates true FH from phenocopies but also strengthens the individualized management of lipid disorders. By enabling therapy to target the specific affected pathway, it represents an important step toward precision medicine and improved patient outcomes. Data sharing initiatives like PerMedFH further improve variant interpretation and clinical utility.

**Supplementary Information:**

The online version contains supplementary material available at 10.1007/s11883-026-01435-x.

## Introduction

Familial Hypercholesterolemia (FH) is a monogenic, autosomal semi-dominant disorder most commonly caused by pathogenic variants in *LDLR*, *APOB*, or *PCSK9*. The heterozygous form (HeFH) affects approximately 1 in 300 individuals, whereas the homozygous form (HoFH), occurring in about 1 in 300,000–400,000 individuals, manifests with a far more severe phenotype [1, 2]. However, several other lipid disorders can clinically mimic the FH phenotype in either the heterozygous or homozygous forms. These include rare autosomal recessive conditions such as sitosterolemia, lysosomal acid lipase deficiency (LALD), and autosomal recessive hypercholesterolemia (ARH). Additionally, in 2013, an *APOE* one–amino acid deletion [*APOE* p.(Leu167del)] was linked to an HeFH-like phenotype [3] .

With the increasing accessibility of expanded genetic testing panels, more cases of these phenocopies have been identified. In 2018, an expert panel recommended the implementation of an eight-gene next-generation sequencing (NGS) panel for the diagnosis of FH and related FH-like disorders to capture this broader genetic spectrum [4]. Since these disorders can be clinically indistinguishable from HeFH or HoFH, genetic testing plays a crucial role in identifying the underlying defect. Accurate molecular diagnosis allows treatment to be tailored to the specific pathway affected, enabling more personalized management and improving clinical outcomes.

## ClinVar – a Public Database

**Fig. 1:**
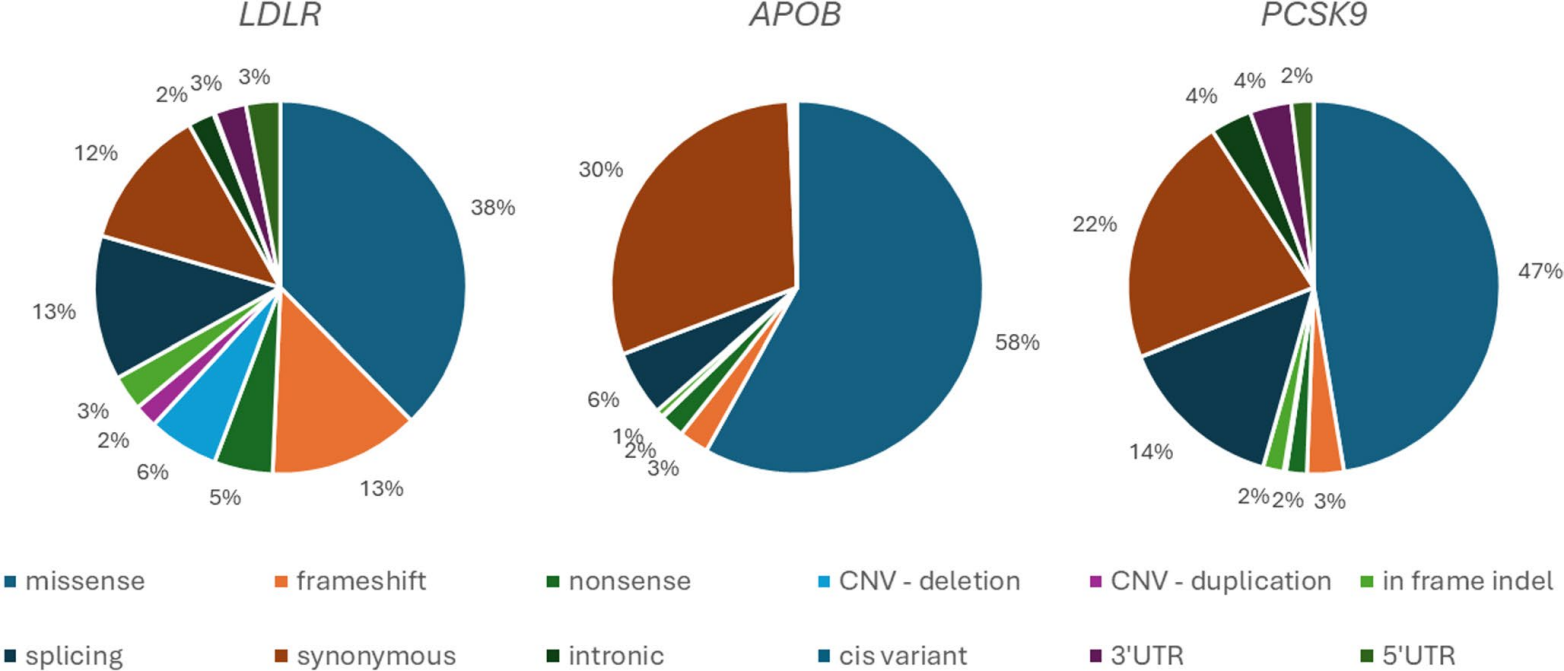
*LDLR*, *APOB* and *PCSK9* variants submitted to ClinVar [9] by variant type

As of December 2025, the ClinVar database lists over 10,800 variants in the *LDLR*, *APOB*, and *PCSK9* genes (Table 1; Fig. 1). In general, these variants have been submitted to ClinVar associated with FH (or hypercholesterolemia phenotypes), however variants in *APOB* and *PCSK9* are not always associated exclusively with FH, since some may cause hypobetalipoproteinemia or other forms of hypocholesterolemia. In ClinVar for *APOB*, over 60% of variants have been wrongly submitted as associated with both hypercholesterolemia and hypocholesterolemia phenotypes, while in *PCSK9* that number is under 10%. It is of extreme importance to assess if the entry disease is the correct one when submitting a variant to ClinVar or when consulting ClinVar. Missense variants are the most frequent type across all three genes—representing 38% in *LDLR*, 58% in *APOB*, and 47% in *PCSK9*—followed by frameshift and splicing variants in *LDLR* (both 13%). Synonymous variants also account for a considerable share in *APOB* (30%) and *PCSK9* (22%) (Fig. 1).

**Table 1:** LDLR, APOB and PCSK9 varianta submitted to ClinVar [9] by classification

Classification	LDLR	APOB*	PCSK9	Total
Pathogenic/Likely pathogenic	2,208	238	18	2,464
Benign/ likely benign	991	1,845	555	3,391
Uncertain significance	1,128	1,822	719	3,669
Conflicting classifications of pathogenicity	313	777	146	1,236
Classification not provided	38	7	5	50
Total	4,678	4,689	1,443	10,810

*most variants submitted associated to hyper and hypo phenotypes

Accurate and standardized classification of these variants is essential for reliable FH genetic testing. Although ClinVar presents aggregated variant classifications, not all submitters follow the same classification algorithms and might reach contradictory conclusions. To address this need, the Clinical Genome Resource (ClinGen), FH Variant Curation Expert Panel (VCEP) has developed specific guidelines for interpreting *LDLR* variants [5], with similar frameworks for other FH-causing genes currently under development. ClinGen has been approved by the Food and Drug administration (FDA) for variant classification, being the first regulatory-grade human variant database, underscoring the importance of a consistent approach, collaboration and sharing of knowledge to improve healthcare.

Of the 4,678 *LDLR* variants reported in ClinVar, 503 have been expert-reviewed by the FH VCEP receiving a three-star classification, one of the highest status for classification evidence (expert review). Among these, 191 are classified as Pathogenic or Likely Pathogenic, 275 as Variants of Uncertain Significance (VUS), and 37 as Likely Benign or Benign [6]. For *LDLR* variants not yet reviewed by the FH VCEP, 1,110 (25%) are nonsense, frameshift, or large deletion variants, generally recognized as null alleles that typically require no further functional characterization for classification. Most of these have been submitted independently by multiple laboratories with consistent interpretations. However, 831 *LDLR* variants are classified as VUS, and an additional 336 show conflicting or missing classifications—together representing another 25% of all *LDLR* entries in ClinVar. VUS or conflicting variants need further evidence to be reclassified. This substantial proportion of VUS underscores the need for comprehensive functional studies and greater sharing of case-level and co-segregation data to achieve consensus and improve variant interpretation. Several large-scale efforts, including the *permedfh.eu* [7] and *fh-early.eu* [8] projects, are actively working to achieve comprehensive functional characterization of VUS, further strengthening data quality and clinical utility.

### FH Causing Genes

#### LDLR

A total of 4,678 *LDLR* variants associated with FH have been reported in ClinVar (Table 1; Fig. 1, Supplementary Table 1). However, not all *LDLR* variants result in the same clinical phenotype. Variants that lead to null alleles—producing no functional LDL-receptor (LDLR) protein or conferring less than 10% residual LDLR activity—are associated with a markedly more severe phenotype and a substantially increased risk of premature coronary heart disease [10]. In contrast, variants that retain between 10% and 70% of receptor activity give rise to heterogeneous phenotypes, with disease severity influenced by both residual receptor function and lifestyle factors [10–12].

Although the classic five-class functional classification of *LDLR* variants in FH—based on defects in receptor synthesis (class 1, null), transport (class 2), binding (class 3), internalization (class 4), or recycling (class 5) — has seen limited use in recent years, emerging functional studies have revived it, along with a proposed class 6 affecting transmembrane domain insertion [13]. This system, originally described by Goldstein and Brown [14], better delineates specific defects to inform prognosis, statin responsiveness, and precision medicine in FH [15]. For example, patients with receptor-recycling defects often respond well to statin therapy—which increases LDLR expression and overcomes the LDLR metabolic defect—without requiring combination drug regimens, provided these patients maintain appropriate lifestyle modifications.

To date, only about 20% of *LDLR* variants have been experimentally characterized. However, recent advances in high-throughput functional assays have greatly accelerated variant interpretation compared with the classical flow cytometry–based reference methods. In the study by Graça et al. 2024 [12], 50 *LDLR* variants were newly characterized, and a 96-sample high-throughput pipeline was optimized and validated against the reference method, demonstrating highly concordant results. This approach captures the full LDLR functional cycle, though binding and internalization are assessed together. Similarly, Islam et al. developed a semi-automated high-throughput method using a biologically relevant model (HepG2 cells), allowing large-scale variant analysis with reduced manual workload. Using this methodology, 350 variants were functionally analysed, generating critical data that have also been integrated with UK Biobank genetic and phenotypic information [10]. More recently, a comprehensive high-throughput functional approach providing near-complete coverage of the *LDLR* coding sequence has been reported [16]. This technique employs multiplexed deep mutational scanning, combining barcoded variant libraries with quantitative cellular assays of LDL uptake and LDLR surface expression for about 17,000 missense variants. The resulting sequence–function maps capture established *LDLR* biology, reveal domain-specific functional constraints, and supply quantitative functional scores correlated with lipid phenotypes and cardiovascular risk across population cohorts. These assays, although need further optimization, are a promising tool for functional characterization of LDLR variants. These assays are being developed for other lipid genes.

Functional data from these and related studies are now being consolidated within the *permed.eu* database [17], which integrates functional, and classification datasets. This resource aims to enhance variant interpretation, facilitate data sharing, and advance precision medicine approaches for FH and related lipid disorders.

### APOB

A total of 4,689 *APOB* variants have been submitted to ClinVar (Table 1; Fig. 1 and Supplementary Table 2). Over 90% were submitted in association with FH, hypercholesterolemia or related cardiovascular phenotypes alone or in combination with other disorders, like hypobetalipoproteinemia. However, a substantial proportion of these variants (61%) have been incorrectly annotated as associated with both hypercholesterolemia and hypocholesterolemia. This misclassification complicates variant interpretation, hampers clinical decision-making, and confounds the apparent contribution of *APOB* to FH in public databases.

Historically, the molecular diagnosis of FH due to *APOB* variants has been restricted to the regions of the gene that were thought to be important for LDL binding. For many years, only a small fragment of exon 26 was screened, encompassing the classical *APOB* variant c.10580G > A, p.(Arg3527Gln), which disrupts LDL binding to the LDLR. Subsequently, a fragment of exon 29 was also incorporated into routine diagnostic protocols [18, 19]. This targeted approach, although pragmatic, inherently biased the spectrum of identified *APOB* variants towards a limited region of the gene and reinforced the concept of a narrow LDLR-binding “hotspot”.

With the expansion of functional studies and the introduction of NGS panels, a broader spectrum of *APOB* variants has emerged. Several missense variants located outside the traditionally screened regions, including p.(Arg1164Thr) in exon 22, and p.(Arg3059Cys), p.(Lys3394Asn), and p.(Thr3826Met) in exon 26, have been shown to reduce LDL binding and/or internalization in functional assays to a degree comparable to p.(Arg3527Gln) [20–23]. In addition, an in-frame deletion of 1 aminoacid in exon 29, p.(Gln4494del), was functionally characterized in 2018 and shown to impair LDL binding and LDLR internalization, further expanding the spectrum of pathogenic *APOB* variants beyond the canonical region, and also the type of *APOB* variants causing FH [21]. Together, these studies demonstrate that functionally relevant *APOB* variants are not confined to a single domain and that pathologic effects can arise from alterations distributed across the protein. However, the polygenic nature of the *APOB* gene cannot be forgotten and functional studies are essential for confirming decreased binding ability.

A defining feature of *APOB*-associated FH is its reduced penetrance and variable expressivity when compared with *LDLR*-associated FH, a pattern consistently observed across cohorts [18, 24, 25]. Even the classical p.(Arg3527Gln) variant shows incomplete penetrance within families, with some carriers displaying only modest LDL elevations or near-normal lipid profiles [18, 26]. This characteristic should be regarded as an intrinsic biological feature of *APOB*-related FH rather than as evidence against pathogenicity [27].

Several factors likely contribute to this reduced penetrance, including the quantitative nature of the apoB100–LDLR interaction, partial preservation of *LDLR* uptake, and modulation by polygenic and environmental factors. In contrast to *LDLR* variants, which often result in marked reductions in receptor-mediated LDL clearance, *APOB* variants typically impair ligand–receptor binding to a variable degree, leading to a more graded phenotypic spectrum [20–22].

This broader mutational and phenotypic landscape can now be interpreted in light of major structural breakthroughs published in 2024 and 2025 [28, 29]. The structure of apoB100 on native LDL particles was resolved using an integrative cryo-EM/AlphaFold-based approach, revealing a large N-terminal globular domain and an extended amphipathic β-sheet “belt” wrapping around the LDL particle surface [29]. Shortly thereafter, cryo-EM structures of apoB100 bound to LDLR defined two distinct interaction interfaces between LDLR and apoB100 and mapped several FH-associated *APOB* variants directly to these interfaces [28].

Together, these studies provide a mechanistic framework supporting the concept that functionally relevant *APOB* variants may occur across multiple regions of the protein, and not only within the historically screened LDLR-binding hotspot. This is illustrated by the distributed mapping of classical variants such as p.(Arg3527Gln), p.(Arg3507Trp) and p.(Arg3558Cys) along the apoB100 β-belt, by the identification of an additional LDLR-interacting interface involving the apoB100 N-terminal domain [28], and by the structural localization of functionally validated variants such as p.(Arg1164Thr) and p.(Gln4494del) [29]. This structural model is also consistent with indirect disruption of apoB100 architecture and long-range conformational interactions, as observed for variants such as p.(Thr3826Met), which lies outside the classical LDLR-binding region yet shows clear functional impairment.

Despite these conceptual, structural and methodological advances, functional characterization of *APOB* variants remains limited. To date, functional validation has been performed only in a small number of cases, demonstrating impaired LDL binding for variants located both within and outside the classical binding region [20–23]. Unlike *LDLR*, functional assessment of *APOB* variants relies on patient-derived serum and LDL particles, which substantially limits scalability and high throughput. This requirement represents a major bottleneck for systematic functional validation and partly explains the slower progress in *APOB* variant characterization compared with *LDLR*.

### PCSK9

ClinVar includes 1,478 variants in *PCSK9* but the majority have not been proven to cause FH (Table 1; Fig. 1 and Supplementary Table 3). In fact, only 18 variants in ClinVar have a Likely Pathogenic/Pathogenic classification and two of those are not FH causing since they lead to premature a stop codon Although pathogenic *PCSK9* variants are found in only a small number of FH patients globally, the discovery of the first *PCSK9* gain-of-function (GOF) variants responsible for FH in French families by Abifadel et al. in 2003 [30], uncovered a new cause of FH and a new therapeutic target that revolutionized the treatment of hypercholesterolemia.

The identification through a genetic approach of linkage analysis and positional cloning of the first two GOF pathogenic variants in the *PCSK9* gene in French families, p.(Ser127Arg) and p.(Phe216Leu), has been pivotal in understanding PCSK9 implication in cholesterol metabolism, hypercholesterolemia and their cardiovascular complications [30–32]. This finding was quickly reinforced by the discovery of a third GOF pathogenic variant, p.(Asp374Tyr), in a Utah family with a severe phenotype of FH, previously linked to the 1p32 region [33].

It was recently shown that the p.(Ser127Arg) variant accounts for 67% of *PCSK9* GOF variants in France, due to a founder effect. Few other carriers of this variant have been reported in South Africa and Norway. It is estimated that the probable common ancestor, who first carried this pathogenic variant, lived around 775 years ago [34].

The p.(Asp374Tyr) was also found in three families from Norway and three from England [35–37]. Another variant in the same codon, p.(Asp374His), was identified in Portugal in 4 unrelated patients showing a very severe phenotype with premature CHD in all affected adult individual [38] and the functional characterization showed a similar cellular effect as p.(Asp374Tyr) [39]. Other GOF variants in PCSK9 have been reported in patients with hypercholesterolemia in different parts of the world, in Europe, USA, Africa and Asia [40–44].

Functional studies, cellular and mouse models, were performed by several teams and deciphered the role of PCSK9 and the impact of its GOF variants, causing hypercholesterolemia by lowering hepatic LDLR levels on cell surface in a non-catalytic way [45]. PCSK9 forms a complex with the LDL receptor and LDL, which is internalized and transported to endosomes. In this compartment, PCSK9 remains bound to the LDL receptor, directing the receptor to lysosomal degradation instead of recycling back to the cell surface. A recent study by Guan YY. et al. [46] elucidated the exact mechanisms by which PCSK9 facilitates the degradation of the LDLR by preventing SNX17-(sorting nexin 17) mediated recycling of the LDL receptor. Normally, acidic pH causes the extracellular domain of the LDL receptor to undergo a conformational change, which facilitates its interaction with SNX17 via the intracellular domain. The conformational shift of the LDL receptor extracellular domain from the cell surface to the acidic environment of the endosome induces a conformational change in its intracellular domain. This modification releases LDLRAP1 (low-density lipoprotein receptor adaptor protein 1) from the NPxY motif and recruits SNX17 to the same motif for endocytic sorting.When PCSK9 binds to LDL receptor, it prevents the acidic pH-induced conformational change of the extracellular domain and its interaction with SNX17 via the intracellular domain. As a result, the LDL receptor protein accumulates in the endosome and is ultimately degraded in the lysosome [46]. This process reduces the number of LDLRs available on hepatocytes, leading to impaired LDL clearance and elevated blood LDL-C levels, and is exacerbated particularly in carriers of GOF variants of *PCSK9* [35, 45, 47].

GOF variants affect mature PCSK9 in several ways: they can reduce autocatalytic cleavage and decrease secretion (e.g. p.(Ser127Arg)), enhance PCSK9 stability by preventing furin-mediated inactivation either completely p.(Arg218Ser) or partially p.(Phe216Leu), or increase binding affinity to the LDL receptor and promote its lysosomal degradation p.(Asp374Tyr). Moreover, an increase in apoB-100 lipoprotein production was reported through in vivo studies in two French carriers of the p.(Ser127Arg) pathogenic variant [48–50].

The molecular basis of *PCSK9* variants is complex. Among GOF variants some pathogenic variants are very rare, while others are more frequent. Several of these variants have been functionally studied and showed association with cardiovascular disease (CVD) [51]. In contrast, *PCSK9* loss-of-function (LOF) variants, notably p.(Tyr142*) and p.(Cys679*) identified later in 2005, are linked to hypocholesterolemia and a reduced risk of CVD [52].

Numerous VUS have been reported and require further exploration. In addition to familial segregation analysis, there is a pressing need for functional tests or rapid methods to assess these variants and understand their impact on hypercholesterolemia and CVD. Protein studies to evaluate the role of PCSK9 as a possible biomarker could also be interesting; however, ELISA measurements require standardization to ensure consistency and reliability in results.

Even though the major challenge for in silico prediction tools is to determine whether a PCSK9 variant affecting the structure or conformation of the protein, is a GOF variant causing FH or a LOF variant, major progress has been made in elucidating PCSK9’s crystal and 3D structures. The development and the use of bioinformatic and structure predictive tools, such as AlphaFold for example, may provide significant support. Docking tools that enable the study of interactions between LDLR and PCSK9 domains are helpful for exploring some variants in these regions. Future advancements in these strategies, helped by AI techniques, such as machine learning and deep learning, are promising in evaluating the exact impact of *PCSK9* variants [53].

Further protein studies to evaluate the role of PCSK9 as a possible biomarker could also be interesting; however, ELISA measurements require standardization to ensure consistency and reliability in results.

## Genes Associated to FH – other Genes Involved in the LDLR Pathway

### APOE

ApoE plays an important role in the VLDL-IDL-LDL metabolic cascade, by binding to hepatic receptors, including the LDLR, and mediating the uptake of IDL and other remnant lipoproteins. Within the field of lipid disorders, *APOE* is best known as the gene responsible for familial dysbetalipoproteinemia [54]. In this disorder, homozygosity for the *APOE* ε2 variant or heterozygosity for certain rare dominant missense variants is associated with markedly increased total cholesterol and triglyceride in plasma, due to increased IDL particles. Familial dysbetalipoproteinemia develops later in life, with a second metabolic ‘hit’ (e.g. obesity, diabetes mellitus) required to manifest the lipid phenotype [55]. However, there are some clinical FH patients presenting with *APOE* ε2 homozygosity and no other genetic cause for their hypercholesterolemia [38]. Genetic testing allows a correct diagnosis for these patients.

In contrast, a 3-nucleotide deletion of codon 167 of *APOE*, p.(Leu167del), results in an autosomal dominant hypercholesterolemia phenotype indistinguishable from FH [56]. Initially, this variant was identified in isolated cases of splenomegaly with sea-blue histiocytosis and hyperlipidemia. Subsequent studies showed that carriers of p.(Leu167del) did not exhibit features of dysbetalipoproteinemia and instead had hypercholesterolemia [3]. Cenarro and colleagues studied 288 individuals with clinical FH but without a pathogenic variant in *LDLR*, *APOB*, or *PCSK9*, and identified nine individuals (3.1%) who were carriers of *APOE* p.(Leu167del), with clear co-segregation of hypercholesterolemia with this deletion, in affected families [57]. The mechanism by which *APOE* p.(Leu167del) results in FH appears to involve enhanced cellular uptake of very-low density lipoprotein (VLDL) particles containing the mutant apoE. Functional studies have shown that VLDL carrying the APOE p.Leu167del variant exhibits increased uptake in heterologous cells, leading to down-regulation of *LDLR* expression and reduced LDL clearance, ultimately resulting in the accumulation of LDL in plasma [57]. A recent study examined the frequency of *APOE* p.(Leu167del) in different populations [58]. Overall, *APOE* p.(Leu167del) is found in approximately 1 in 12,000 individuals in the general population, but 2.5% of FH, although data are lacking in East Asian and Middle Eastern populations. In a Spanish cohort, carriers of *APOE* p.(Leu167del) (*n* = 29) had higher median triglyceride (167 vs. 115 md/dL) and high density lipoprotein (HDL)-cholesterol levels (61.0 vs. 54.8 mg/dL), and lower LDL-cholesterol levels (255 vs. 281 mg/dL), compared with individuals with an FH-causing *LDLR*, *APOB*, or *PCSK9* variant (*n* = 339). Carriers of *APOE* p.(Leu167del) also had a lower prevalence of tendon xanthomata (3.4% vs. 27% in *LDLR*/*APOB*/*PCSK9* FH), which could relate to the lower LDL-cholesterol concentration and younger age of this group [58].

### LDLRAP1 (Autosomal Recessive Hypercholesterolemia)

Autosomal recessive hypercholesterolemia (ARH) due to bi-allelic variants in the LDLR adaptor protein-1 gene (*LDLRAP1*) presents a very similar phenotype to HoFH due to biallelic *LDLR* variants [59]. In the latest European Atherosclerosis Society consensus on HoFH, variants in *LDLRAP1* are considered a form of HoFH [60]. This form of severe hypercholesterolemia is rare, even among HoFH; of 71 individuals with HoFH in the National Dyslipidemia Registry of the Spanish Atherosclerosis Society, only four (5.6%) had ARH [61]; in the HICC (HoFH, the International Clinical Collaborators) registry, in 404 HoFH due to *LDLR*,* APOB* and *PCSK9* biallelic variants, only twelve (3%) have biallelic variants in *LDLRAP1* [62]. Although the ARH genetic defect is within the LDLR pathway, it is worth noting that LDLRAP1 has a different role in different tissues. For example, in ARH there is normal LDLR activity in fibroblasts, which do not use LDLRAP1 protein to internalize the LDLR: LDL complex. Instead, another adaptor protein, Dab2, mediates internalization in these cells [63]. The liver and lymphocytes do require LDLRAP1 and, with the majority of ARH patients having two null *LDLRAP1* variants, the liver is incapable of internalizing LDL. Treatment that removes LDL from circulation by an LDLR-independent mechanism is very important for these patients.

Worldwide only a very small number of ARH patients have been reported, although there is a higher prevalence in Sardinia, probably due to a founder effect [64, 65].

## FH Phenocopies - it Seems FH but it is not FH

### ABCG5 and ABCG8 (Sitosterolemia)

Several monogenic disorders can clinically mimic FH, presenting with elevated cholesterol, xanthomas and premature cardiovascular disease, despite distinct underlying mechanisms. Among these FH phenocopies, sitosterolemia is one of the most clinically relevant and frequently underdiagnosed conditions. Sitosterolemia is an autosomal recessive disorder caused by loss-of-function variants in *ABCG5* or *ABCG8*, genes encoding sterol transporters responsible for limiting intestinal absorption and promoting biliary excretion of dietary sterols [66, 67]. This model was historically supported by the observation that most reported cases involved biallelic variants, suggesting that near-complete loss of transporter function is required for disease manifestation. However, emerging evidence indicates that heterozygous carriers of *ABCG5* loss-of-function variants may exhibit increased LDL-cholesterol levels and FH-like features, suggesting a potential codominant effect and a broader phenotypic spectrum. These observations challenge the strict classification of sitosterolemia as a purely recessive disorder [68]. Although most cases are caused by biallelic variants in a single gene, a case involving variants in both *ABCG5* and *ABCG8* has been reported, with markedly elevated plasma sitosterol levels confirmed by chromatographic analysis, highlighting the genetic and biochemical heterogeneity of the disease [69].

Clinically, sitosterolemia closely resembles FH and is therefore often misdiagnosed. In a recent report describing 16 cases of sitosterolemia in Ibero-America [70], all patients had initially received a diagnosis of FH, with a substantial delay before the correct diagnosis was established. This delay had important consequences for patient management, resulting in inappropriate lipid-lowering therapy and dietary advice, contributing to cardiovascular complications in several individuals. Notably, in the past three years, an additional 14 cases of sitosterolemia have been reported in Brazil, also in an FH cohort [71], reflecting a marked increase in disease recognition that is largely attributable to the widespread use of multigene next-generation sequencing panels, which enable systematic differentiation between FH and FH-like phenotypes such as sitosterolemia [72, 73].

The pathophysiology of sitosterolemia is characterized by increased intestinal absorption and impaired biliary excretion of plant sterols, leading to their accumulation in plasma and tissues. Because plant sterols are structurally similar to cholesterol, they are incorporated into circulating lipoproteins and are not distinguished from cholesterol by routine lipid assays, explaining the FH-like lipid profile. In addition, excess plant sterols may disrupt cholesterol homeostasis and LDLR regulation, contributing to hypercholesterolemia and atherosclerosis [74–77]. Importantly, this mechanism largely differs from FH, with major therapeutic implications, including distinct diet approaches and treatment targeting sterol absorption, such as ezetimibe, are particularly effective, whereas statins alone are often insufficient.

### LIPA (Lysosomal Acid Lipase Deficiency)

Lysosomal Acid Lipase Deficiency (LALD) is an autosomal recessive lysosomal storage disorder with two distinct presentations. The most severe infant-onset form that leads to no LAL activity, formerly known as Wolman Syndrome, and the milder adult-onset version where LAL still holds some activity, formerly known as cholesterol ester storage disease [78]. In this milder form, the most common cause is a synonymous variant c.894G > A p.(Gln298=) (E8SJ, exon 8 splice junction variant), that impairs splicing and leads to loss of amino acids 254–277.

The LALD phenotype with high cholesterol can be mistaken for FH, although sometimes also presents with mild to high triglycerides. One of the key symptoms of LALD is an altered liver function (e.g. elevated aminotransferase levels) that can be missed in the milder adult-onset form, especially if the patient is on statins. As such, in the last decade patients with a clinical diagnosis of FH were screened for *LIPA* gene variants, and some were identified with LALD. Namely, in 2015 the US identified one family [79], in 2017 Portugal reported four cases from three families [80], and in 2022 Slovenia presented three cases from two families [81], all from presumably clinical FH cases.

Treatment that increases the LDL accumulation in the liver could be damaging for these patients that cannot esterify cholesterol into cholesterol esters because cholesterol can stay in the liver and overload the lysosomes [80]. Due to its severe atherogenic phenotype [82], there are patients with LALD being treated with statins but these patients should have a strict follow up. PCSK9 inhibitors are not recommend for these patients for the same reason, but ezetimibe can be used safely. Since its approval in 2015, there is a specific enzyme replacement therapy for these patients, a human recombinant LAL enzyme (sebelipase alfa), that greatly improves their hepatic and lipid profiles and even improves survival in the most severe infant-onset form [83–85].

## Summary

Familial hypercholesterolemia (FH) remains underdiagnosed and undertreated worldwide, leaving affected individuals at high risk of preventable cardiovascular complications. Expansion of genetic diagnosis to an 8-gene panel—with complete sequencing of the three core FH-causing genes (LDLR, APOB, PCSK9) plus FH-associated and phenocopy genes—has improved accurate patient identification.

Incorporating functional assays to assess variant effects on cholesterol metabolism further refines disease severity categories and enables tailored therapies, advancing precision medicine for FH. Accurate variant classification—using ACMG guidelines, ClinGen gene specifications, and functional assays—is crucial to reduce variants of uncertain significance.

Although much is known about FH’s genetic determinants, with substantial advances improving understanding and management of its diverse phenotypes and genotypes, significant challenges remain. The major gap lies in clinical diagnosis, requiring targeted strategies such as expanded screening in high-risk populations, increased medical awareness of FH’s clinical and genetic features, and affordable genetic testing to promote early detection. As emphasized in the European Cardiovascular Plan, universal FH screening in paediatric populations addresses underdiagnosis and enables timely intervention during the optimal window for cardiovascular prevention. Early, lifelong.

therapy initiated in childhood offers cardiovascular event-free lives for all individuals with FH.

While clinical identification remains the central challenge in primary care, advances in genetic diagnostics and initiatives like permedfh.eu and fh-early.eu reduce variants of uncertain significance, improving diagnostic accuracy, prognostic assessment, and personalized treatment.

## Conclusions and Future Perspectives

Despite major advances in genetic diagnostics and variant characterization, familial hypercholesterolemia remains largely underdiagnosed, representing the main barrier to effective cardiovascular prevention. Expanding systematic screening—particularly in pediatric and high-risk populations—together with improving access to genetic testing and increasing clinical awareness, are key priorities.

Ongoing efforts to integrate functional data and harmonize variant interpretation will further enhance diagnostic accuracy and support personalized treatment. Collaborative initiatives such as *permedfh.eu* and *fh-early.eu* are instrumental in advancing this field, paving the way for more effective, precision-based management of FH.

## Key References

- Islam MM, Tamlander M, Hlushchenko I, Ripatti S, Pfisterer SG. Large-Scale Functional Characterization of Low-Density Lipoprotein Receptor Gene Variants Improves Risk Assessment in Cardiovascular Disease. JACC Basic to Transl Sci. 2025;10:170–83. 10.1016/j.jacbts.2024.10.006
  - ○ The study provides large-scale functional characterization for 315 LDLR variants, showing that residual receptor activity influences LDL-C levels, cardiovascular risk, and the use of lipid-lowering and combination therapy, thereby enhancing early diagnosis, risk stratification, and treatment selection in FH
- Graça R, Zimon M, Alves AC, Pepperkok R, Bourbon M. High-Throughput Microscopy Characterization of Rare LDLR Variants. JACC Basic to Transl Sci. 2023;8:1010–21. 10.1016/J.JACBTS.2023.03.013
  - ○ This study describes a high-throughput microscopy approach for the functional characterization of rare LDLR variants, providing quantitative evidence to support variant classification and improve the diagnosis of familial hypercholesterolemia
- Tabet DR, Coté AG, Lancaster MC, Weile J, Rayhan A, Fotiadou I, et al. The functional landscape of coding variation in the familial hypercholesterolemia gene LDLR. Science. 2025; 10.1126/SCIENCE.ADY7186
  - ○ A multiplexed in vitro assay was developed to perform a large-scale functional characterization of nearly all possible (almost 17,000) LDLR missense variants. The structure-function maps produced offer mechanistic insights and will be an important tool for variant interpretation
- Rodríguez-Jiménez C, de la Peña G, Sanguino J, Poyatos-Peláez S, Carazo A, Martínez-Hernández PL, et al. Identification and Functional Analysis of APOB Variants in a Cohort of Hypercholesterolemic Patients. Int J Mol Sci. 2023;24. 10.3390/IJMS24087635
  - ○ This study identifies and functionally characterizes APOB variants in hypercholesterolemic patients, providing functional evidence that supports variant interpretation and improves the genetic diagnosis of familial hypercholesterolemia
- Reimund M, Dearborn AD, Graziano G, Lei H, Ciancone AM, Kumar A, et al. Structure of apolipoprotein B100 bound to the low-density lipoprotein receptor. Nature. 2025;638:829–35. 10.1038/S41586-024-08223-0
  - ○ Cryo-electron microscopy was used to reveal how apolipoprotein B100 binds the LDL receptor through two distinct interfaces, providing key insights into LDL particle recognition and cholesterol metabolism
- Berndsen ZT, Cassidy CK. The structure of apolipoprotein B100 from human low-density lipoprotein. Nature. 2024;638:836–43. 10.1038/s41586-024-08467-w
  - ○ This study elucidates the high-resolution structure of human apolipoprotein B100 within low-density lipoprotein particles, providing key structural insights that enhance the understanding of LDL assembly, receptor interactions, and the molecular impact of APOB variants in dyslipidemia
- Abifadel M. PCSK9 in Familial Hypercholesterolemia and Beyond. JAMA Cardiol. American Medical Association; 2025;10:755–6. 10.1001/JAMACARDIO.2025.1408
  - ○ This article highlights how the discovery of pathogenic variants of PCSK9 in familial hypercholesterolemia sparked research that led to the development of highly effective PCSK9 inhibitors, revolutionizing cholesterol treatment and improving patient outcomes
- Abifadel M. Scientific journey from PSCK9 discovery in familial hypercholesterolaemia. Nat Rev Cardiol. 2025;22:387–8. 10.1038/s41569-025-01147-w
  - ○ The article reflects on the scientific journey from the discovery of the role of PCSK9 in familial hypercholesterolaemia to its profound impact on understanding cholesterol metabolism and developing transformative therapies targeting PCSK9, while emphasizing the ongoing need to ensure equitable access to these drugs
- Azar Y, Ludwig TE, Le Bon H, Strøm TB, Bluteau O, Di-Filippo M, et al. The singular French PCSK9-p.Ser127Arg gain-of-function variant: A significant player in cholesterol levels from a 775-year-old common ancestor. Atherosclerosis. 2024;399:118596. 10.1016/J.ATHEROSCLEROSIS.2024.118596
  - ○ The French PCSK9-p.Ser127Arg gain-of-function variant was traced to a common ancestor that lived around 775 years ago and highlights its significant impact on cholesterol levels in carriers
- Guan Y, Liu X, Yang Z, Zhu X, Liu M, Du M, et al. PCSK9 Promotes LDLR Degradation by Preventing SNX17-Mediated LDLR Recycling. Circulation. 2025;151:1512–26. 10.1161/CIRCULATIONAHA.124.072336
  - ○ The study shows that PCSK9 drives LDLR degradation by blocking SNX17-mediated recycling, and patients with FH carrying LDLR recycling defects are resistant to PCSK9 inhibitors, highlighting the need for genetic diagnosis and alternative LDLR-independent therapies
- Bello-Álvarez D, Cenarro A, Bea AM, Marco-Benedí V, Jarauta E, Ortiz-Palma P, et al. APOE p.(Leu167del) variant in hypercholesterolemia: Prevalence & phenotypic expression. J Clin Lipidol. 2025; 10.1016/j.jacl.2025.09.016
  - ○ This study confirms that the APOE p.(Leu167del) variant is associated with hypercholesterolemia, though with lower LDL-C than FH caused by LDLR, APOB, or PCSK9 variants, and supports its inclusion in FH genetic screening, particularly in Caucasian populations
- Sánchez-Hernández RM, Ibarretxe D, Fuentes Jiménez F, Martínez-Hervás S, Blanco-Echevarría A, Cortés Rodríguez B, et al. Homozygous Familial Hypercholesterolemia in Spain: Data From Registry of the Spanish Atherosclerosis Society. J Clin Endocrinol Metab. 2025;110:2280–7. 10.1210/CLINEM/DGAE784
  - ○ Data from the Spanish Dyslipidemia Registry and molecular diagnoses performed for FH in Spain between 1996 and 2015 highlights a higher-than-expected HoFH prevalence, with over half of genetically confirmed patients not meeting classical clinical criteria, and a phenotype ranging from severe to mild, underscoring the importance of molecular testing and improved diagnostic procedures for HoFH diagnosis.
- Reijman MD, Tromp TR, Hutten BA, Hovingh GK, Blom DJ, Catapano AL, et al. Cardiovascular outcomes in patients with homozygous familial hypercholesterolaemia on lipoprotein apheresis initiated during childhood: long-term follow-up of an international cohort from two registries. Lancet Child Adolesc Health. 2024;8:491–9. 10.1016/S2352-4642(24)00073-7
  - ○ This study reports long-term cardiovascular outcomes in patients with homozygous familial hypercholesterolemia who initiated lipoprotein apheresis during childhood, demonstrating sustained cardiovascular benefit and supporting early intensive treatment to reduce lifelong cardiovascular risk
- Alves AC, Chora JR, Miranda B, Medeiros AM, Graça R, Bañares VG, et al. Sitosterolemia in Iberoamerican countries: 16 new cases and phenotype genotype analysis. J Clin Lipidol. 2025;19:1715–24. 10.1016/J.JACL.2025.08.020
  - ○ The study reports 16 cases of molecularly confirmed of sitosterolemia in Iberoamerican countries with a detailed analysis of the genotype–phenotype relationships, and highlights frequent misdiagnosis as familial hypercholesterolemia, the need to incorporate ABCG5/8 into genetic testing panels and to identify clinical signs early, to improve outcomes and prevent complications.
- Nomura A, Emdin CA, Won HH, Peloso GM, Natarajan P, Ardissino D, et al. Heterozygous ATP-binding Cassette Transporter G5 Gene Deficiency and Risk of Coronary Artery Disease. Circ Genomic Precis Med. Lippincott Williams and Wilkins; 2020;13:417. 10.1161/CIRCGEN.119.00287
  - ○ This study challenges the view of sitosterolemia as a strictly recessive disorder by showing that heterozygous ABCG5 loss-of-function carriers have elevated LDL-C and a two-fold increased risk of coronary artery disease. These findings highlight the contribution of partial defects in sterol transport to cardiovascular risk and broaden the clinical relevance of this condition
- Fornengo P, Ferro A, Fagoonee S, Rinaudo E, Amione C, Durazzo M. Lysosomal acid lipase deficiency: The forgotten link between liver and cardiovascular disease. World J Cardiol. 2025;17. 10.4330/WJC.V17.I11.111292
  - ○ A comprehensive review of lysosomal acid lipase deficiency, focusing on the late-onset form, cholesteryl ester storage disease, and emphasizes its link to premature atherosclerosis and cardiovascular disease, its frequent under-recognition, and the importance of early diagnosis, treatment, and clinician awareness. Thus, prompt identification and treatment are essential, and greater awareness of this condition among clinicians is required
- Vijay S, Evans J, Lacaille F, Abel F, Heras J de las. Survival, growth, and safety findings in patients with rapidly progressive, infantile-onset LAL-D: Results from the international LAL-D registry. Mol Genet Metab. 2025;146:109290. 10.1016/j.ymgme.2025.109290
  - ○ The study of 29 infants with rapidly progressive LAL-D from the International LAL-D Registry demonstrates that treatment with sebelipase alfa improves survival, growth, and metabolic outcomes, with generally mild and mostly resolved adverse events, while highlighting the need for future research to establish strategies for managing the disease as patients grow into adolescence and adulthood.

## Supplementary Information

Below is the link to the electronic supplementary material.

Supplementary Material 1

Supplementary Material 2

Supplementary Material 3

## Data Availability

No datasets were generated or analysed during the current study.

## References

[1] Beheshti SO, Madsen CM, Varbo A, Nordestgaard BG. Worldwide Prevalence of Familial Hypercholesterolemia. J Am Coll Cardiol. 2020;75:2553–66. 10.1016/j.jacc.2020.03.057. 32439005 10.1016/j.jacc.2020.03.057

[2] Hu P, Dharmayat KI, Stevens CAT, Sharabiani MTA, Jones RS, Watts GF, et al. Prevalence of Familial Hypercholesterolemia Among the General Population and Patients With Atherosclerotic Cardiovascular Disease: A Systematic Review and Meta-Analysis. Circulation Circulation. 2020;141:1742–59. 10.1161/CIRCULATIONAHA.119.044795. 32468833 10.1161/CIRCULATIONAHA.119.044795

[3] Marduel M, Ouguerram K, Serre V, Bonnefont-Rousselot D, Marques-Pinheiro A, Erik Berge K, et al. Description of a large family with autosomal dominant hypercholesterolemia associated with the APOE p.Leu167del mutation. Hum Mutat Hum Mutat. 2013;34:83–7. 10.1002/HUMU.22215. 22949395 10.1002/humu.22215 PMC3638718

[4] Sturm AC, Knowles JW, Gidding SS, Ahmad ZS, Ahmed CD, Ballantyne CM, et al. Clinical Genetic Testing for Familial Hypercholesterolemia: JACC Scientific Expert Panel. J Am Coll Cardiol. 2018;72:662–80. 10.1016/j.jacc.2018.05.044. 30071997 10.1016/j.jacc.2018.05.044

[5] Chora JR, Iacocca MA, Tichý L, Wand H, Kurtz CL, Zimmermann H, et al. The Clinical Genome Resource (ClinGen) Familial Hypercholesterolemia Variant Curation Expert Panel consensus guidelines for LDLR variant classification. Genet Med Elsevier BV. 2022;24:293–306. 10.1016/J.GIM.2021.09.012. 10.1016/j.gim.2021.09.012 PMC12558601 34906454

[6] Chora J, Hooper A, Ford CG, Kullo I, Bourbon M. Development of gene-specific ACMG/AMP guidelines for the interpretation of APOB and PCSK9 variants in familial hypercholesterolemia. Atherosclerosis Elsevier BV. 2025;407:119569. 10.1016/j.atherosclerosis.2025.119569.

[7] PerMedFH – personalised medicine for familial hypercholesterolaemia. 2025. [cited 2026 Feb 6]. https://permedfh.eu/. Accessed 6 Feb 2026.

[8] FH-EARLY: transforming familial hypercholesterolemia diagnosis.

[9] ClinVar - ClinVar. aggregates information about genomic variation and its relationship to human health. 2025 [cited 2026 Feb 6]. https://www.ncbi.nlm.nih.gov/clinvar/. Accessed 6 Feb 2026.

[10] Islam MM, Tamlander M, Hlushchenko I, Ripatti S, Pfisterer SG. Large-Scale Functional Characterization of Low-Density Lipoprotein Receptor Gene Variants Improves Risk Assessment in Cardiovascular Disease. JACC Basic Transl Sci. 2025;10:170–83. 10.1016/j.jacbts.2024.10.006. 40131152 10.1016/j.jacbts.2024.10.006 PMC11897452

[11] Alves AC, Azevedo S, Benito-Vicente A, Graça R, Galicia-Garcia U, Barros P, et al. LDLR variants functional characterization: Contribution to variant classification. Atherosclerosis Atherosclerosis. 2021;329:14–21. 10.1016/J.ATHEROSCLEROSIS.2021.06.001. 34167030 10.1016/j.atherosclerosis.2021.06.001

[12] Graça R, Zimon M, Alves AC, Pepperkok R, Bourbon M. High-Throughput Microscopy Characterization of Rare LDLR Variants. JACC Basic to Transl Sci. JACC Basic Transl Sci. 2023;8:1010–21. 10.1016/J.JACBTS.2023.03.013. 37719435 10.1016/j.jacbts.2023.03.013 PMC10504398

[13] Ibrahim S, Defesche JC, Kastelein JJP. Beyond the usual suspects: expanding on mutations and detection for familial hypercholesterolemia. Expert Rev Mol Diagn. 2021;21:887–95. 34263698 10.1080/14737159.2021.1953985

[14] Brown MS, Goldstein JL. A receptor-mediated pathway for cholesterol homeostasis. Science (80-). 1986/04/04. 1986;232:34–47. http://www.ncbi.nlm.nih.gov/pubmed/3513311. 10.1126/science.3513311 3513311

[15] Graça R, Alves AC, Zimon M, Pepperkok R, Bourbon M. Functional profiling of LDLR variants: important evidence for variant classification: functional profiling of LDLR variants. J Clin Lipidol 16:516–24. 10.1016/j.jacl.2022.04.005. 10.1016/j.jacl.2022.04.005 35568682

[16] Tabet DR, Coté AG, Lancaster MC, Weile J, Rayhan A, Fotiadou I, Kishore N, Li R, Kuang D, Knapp JJ, Carrero CS, Taverniti O, Axakova A, Castelli JMP, Islam MM, Sowlati-Hashjin S, Gandhi A, Maaieh R, Garton M, Matreyek K, Fowler DM, Bourbon M, Pfisterer SG, Glazer AM, Kroncke BM, Parikh VN, Ashley EA, Knowles JW, Claussnitzer M, Cirulli ET, Hegele RA, Roden DM, MacRae CA, Roth FP. The functional landscape of coding variation in the familial hypercholesterolemia gene LDLR. Science. 2026;391(6787):eady7186. 10.1126/SCIENCE.ADY7186. 10.1126/science.ady7186 PMC12726852 41166440

[17] PerMedFH variant platform – precision drug optimisation for FH. 2025 [cited 2026 Feb 6]. https://permedfh.eu/variant-platform/. Accessed 6 Feb 2026.

[18] Huijgen R, Sjouke B, Vis K, de Randamie JSE, Defesche JC, Kastelein JJP, et al. Genetic variation in APOB, PCSK9, and ANGPTL3 in carriers of pathogenic autosomal dominant hypercholesterolemic mutations with unexpected low LDL-Cl Levels. Hum Mutat. 2012;33:448–55. 10.1002/humu.21660. 22095935 10.1002/humu.21660

[19] Medeiros AM, Alves AC, Bourbon M. Mutational analysis of a cohort with clinical diagnosis of familial hypercholesterolemia: considerations for genetic diagnosis improvement. Genet Med IOP Publishing. 2015;18:1–9. 10.1038/gim.2015.71. 10.1038/gim.2015.71 26020417

[20] Alves AC, Etxebarria A, Soutar AK, Martin C, Bourbon M. Novel functional APOB mutations outside LDL-binding region causing familial hypercholesterolaemia. Hum Mol Genet. 2014;23:1817–28. 10.1093/hmg/ddt573. 24234650 10.1093/hmg/ddt573

[21] Alves AC, Azevedo S, Benito-Vicente A, Etxebarria A, Barros P, Martin C et al. Further function characterization of putative variants in the LDLR. Atherosclerosis. 2018;(submitted for publication).

[22] Motazacker MM, Pirruccello J, Huijgen R, Do R, Gabriel S, Peter J, et al. Advances in genetics show the need for extending screening strategies for autosomal dominant hypercholesterolaemia. Eur Heart J. 2012;33:1360–6. 10.1093/eurheartj/ehs010. 22408029 10.1093/eurheartj/ehs010

[23] Rodríguez-Jiménez C, de la Peña G, Sanguino J, Poyatos-Peláez S, Carazo A, Martínez-Hernández PL, et al. Identification and functional analysis of APOB variants in a cohort of hypercholesterolemic patients. Int J Mol Sci Int J Mol Sci. 2023;24. 10.3390/IJMS24087635. 10.3390/ijms24087635 PMC10142790 37108800

[24] Pullinger CR, Hennessy LK, Chatterton JE, Liu W, Love JA, Mendel CM, et al. Familial ligand-defective apolipoprotein B. Identification of a new mutation that decreases LDL receptor binding affinity. J Clin Invest. 1995;95:1225–34. 10.1172/JCI117772. 7883971 10.1172/JCI117772 PMC441461

[25] Nordestgaard BG, Chapman MJ, Humphries SE, Ginsberg HN, Masana L, Descamps OS, et al. Familial hypercholesterolaemia is underdiagnosed and undertreated in the general population: guidance for clinicians to prevent coronary heart disease: Consensus Statement of the European Atherosclerosis Society. Eur Heart J. 2013;34:3478–90. 10.1093/eurheartj/eht273. 23956253 10.1093/eurheartj/eht273 PMC3844152

[26] Soria LF, Ludwing EH, Clarke HRG, Vega GL, Grundy SM, McCarthy BJ. Association between a specific apolipoprotein B mutation and familial defective apolipoptotein B-100. Proc Natl Acad Sci USA. 1989;86:587–91. 2563166 10.1073/pnas.86.2.587 PMC286517

[27] Vrablik M, Tichý L, Freiberger T, Blaha V, Satny M, Hubacek JA. Genetics of familial hypercholesterolemia: new insights. Front Genet Front Media SA. 2020;11. 10.3389/fgene.2020.574474. 10.3389/fgene.2020.574474 PMC7575810 33133164

[28] Reimund M, Dearborn AD, Graziano G, Lei H, Ciancone AM, Kumar A, et al. Structure of apolipoprotein B100 bound to the low-density lipoprotein receptor. Nat Nat Res. 2025;638:829–35. 10.1038/S41586-024-08223-0. ;TECHMETA=101,28;SUBJMETA=1258,1259,443,535,592,631,75;KWRD=CARDIOVASCULAR+DISEASES,CRYOELECTRON+MICROSCOPY. 10.1038/s41586-024-08223-0 39663455

[29] Berndsen ZT, Cassidy CK. The structure of apolipoprotein B100 from human low-density lipoprotein. Nat 2024 6388051 Nat Publishing Group. 2024;638:836–43. 10.1038/s41586-024-08467-w. 10.1038/s41586-024-08467-w PMC11839476 39662503

[30] Abifadel M, Varret M, Rabès J-P, Allard D, Ouguerram K, Devillers M, et al. Mutations in PCSK9 cause autosomal dominant hypercholesterolemia. Nat Genet. 2003;34:154–6. 10.1038/ng1161. 12730697 10.1038/ng1161

[31] Abifadel M. PCSK9 in Familial Hypercholesterolemia and Beyond. JAMA Cardiol Am Med Association. 2025;10:755–6. 10.1001/JAMACARDIO.2025.1408. 10.1001/jamacardio.2025.1408 40465305

[32] Abifadel M. Scientific journey from PSCK9 discovery in familial hypercholesterolaemia. Nat Rev Cardiol. 2025;22:387–8. . 10.1038/s41569-025-01147-w 40155484 10.1038/s41569-025-01147-w

[33] Timms KM, Wagner S, Samuels ME, Forbey K, Goldfine H, Jammalapati S, et al. A mutation in PCSK9 causing autosomal-dominant hypercholesterolemia in a Utah pedigree. Hum Genet. 2004;114:349–53. 10.1007/s00439-003-1071-9. 14727179 10.1007/s00439-003-1071-9

[34] Azar Y, Ludwig TE, Le Bon H, Strøm TB, Bluteau O, Di-Filippo M, et al. The singular French PCSK9-p.Ser127Arg gain-of-function variant: A significant player in cholesterol levels from a 775-year-old common ancestor. Atherosclerosis Elsevier. 2024;399:118596. 10.1016/J.ATHEROSCLEROSIS.2024.118596. 10.1016/j.atherosclerosis.2024.118596 39500114

[35] Abifadel M, Boileau C. Genetic and molecular architecture of familial hypercholesterolemia. J Intern Med J Intern Med. 2023;293:144–65. 10.1111/JOIM.13577. 36196022 10.1111/joim.13577 PMC10092380

[36] Leren TP. Mutations in the PCSK9 gene in Norwegian subjects with autosomal dominant hypercholesterolemia. Clin Genet. 2004;65:419–22. 10.1111/j.0009-9163.2004.0238.x. 15099351 10.1111/j.0009-9163.2004.0238.x

[37] Sun XM, Eden ER, Tosi I, Neuwirth CK, Wile D, Naoumova RP, et al. Evidence for effect of mutant PCSK9 on apolipoprotein B secretion as the cause of unusually severe dominant hypercholesterolaemia. Hum Mol Genet. 2005;14:1161–9. 10.1093/hmg/ddi128. 15772090 10.1093/hmg/ddi128

[38] Medeiros AM, Alves AC, Miranda B, Chora JR, Bourbon M. Unraveling the genetic background of individuals with a clinical familial hypercholesterolemia phenotype. J Lipid Res J Lipid Res. 2024;65. 10.1016/J.JLR.2023.100490. 10.1016/j.jlr.2023.100490 PMC10832474 38122934

[39] Fasano T, Sun X-M, Patel DD, Soutar AK. Degradation of LDLR protein mediated by gain of function PCSK9 mutants in normal and ARH cells. Atherosclerosis. 2009;203:166–71. 10.1016/j.atherosclerosis.2008.10.027. 19081568 10.1016/j.atherosclerosis.2008.10.027

[40] Abifadel M, Guerin M, Benjannet S, Rabès J-P, Le Goff W, Julia Z, et al. Identification and characterization of new gain-of-function mutations in the PCSK9 gene responsible for autosomal dominant hypercholesterolemia. Atherosclerosis. 2012;223:394–400. 10.1016/j.atherosclerosis.2012.04.006. 22683120 10.1016/j.atherosclerosis.2012.04.006

[41] Di Taranto MD, Benito-Vicente A, Giacobbe C, Uribe KB, Rubba P, Etxebarria A, et al. Identification and in vitro characterization of two new PCSK9 Gain of Function variants found in patients with Familial Hypercholesterolemia. Sci Reports 2017 71. Nat Publishing Group. 2017;7:15282. 10.1038/s41598-017-15543-x. 10.1038/s41598-017-15543-x PMC5681505 29127338

[42] Guo Q, Feng X, Zhou Y. PCSK9 Variants in Familial Hypercholesterolemia: A Comprehensive Synopsis. Front Genet Front Media S A. 2020;11:547103. 10.3389/FGENE.2020.01020/FULL. 10.3389/fgene.2020.01020 PMC7538608 33173529

[43] Huijgen R, Blom DJ, Hartgers ML, Chemello K, Benito-Vicente A, Uribe KB, et al. Novel PCSK9 (proprotein convertase subtilisin Kexin Type 9) variants in patients with familial hypercholesterolemia from Cape Town. Arterioscler Thromb Vasc Biol. 2021;41:934–43. 33147992 10.1161/ATVBAHA.120.314482

[44] Bourbon M, Alves AC, Medeiros AM, Silva S, Soutar AK. Familial hypercholesterolaemia in Portugal. Atherosclerosis. 2008;196:633–42. 2007/09/04. 17765246 10.1016/j.atherosclerosis.2007.07.019

[45] Benjannet S, Rhainds D, Essalmani R, Mayne J, Wickham L, Jin W, et al. NARC-1/PCSK9 and its natural mutants: zymogen cleavage and effects on the low density lipoprotein (LDL) receptor and LDL cholesterol. J Biol Chem. 2004;279:48865–75. 10.1074/jbc.M409699200. 15358785 10.1074/jbc.M409699200

[46] Guan Y, Liu X, Yang Z, Zhu X, Liu M, Du M, et al. PCSK9 Promotes LDLR Degradation by Preventing SNX17-Mediated LDLR Recycling. Circulation. Lippincott Williams Wilkins. 2025;151:1512–26. 10.1161/CIRCULATIONAHA.124.072336;WGROUP:STRING:PUBLICATION. 10.1161/CIRCULATIONAHA.124.072336 40071387

[47] El Khoury P, Elbitar S, Ghaleb Y, Khalil YA, Varret M, Boileau C, et al. PCSK9 Mutations in familial hypercholesterolemia: from a groundbreaking discovery to anti-PCSK9 therapies. Curr Atheroscler Rep Curr Med. 2017;19:49. . 10.1007/S11883-017-0684-8/TABLES/1 10.1007/s11883-017-0684-8 29038906

[48] Essalmani R, Susan-Resiga D, Chamberland A, Abifadel M, Creemers JW, Boileau C, et al. In Vivo Evidence That Furin from Hepatocytes Inactivates PCSK9. J Biol Chem Elsevier. 2011;286:4257–63. 10.1074/JBC.M110.192104. 10.1074/jbc.M110.192104 PMC3039354 21147780

[49] Lagace TA, Curtis DE, Garuti R, McNutt MC, Sahng WP, Prather HB, et al. Secreted PCSK9 decreases the number of LDL receptors in hepatocytes and in livers of parabiotic mice. J Clin Invest J Clin Invest. 2006;116:2995–3005. 10.1172/JCI29383. [cited 2024 Jan 1];. 17080197 10.1172/JCI29383 PMC1626117

[50] Ouguerram K, Chetiveaux M, Zair Y, Costet P, Abifadel M, Varret M, et al. Apolipoprotein B100 metabolism in autosomal-dominant hypercholesterolemia related to mutations in PCSK9. Arterioscler Thromb Vasc Biol. 2004;24:1448–53. 15166014 10.1161/01.ATV.0000133684.77013.88

[51] Abifadel M, Rabès J-P, Devillers M, Munnich A, Erlich D, Junien C, et al. Mutations and polymorphisms in the proprotein convertase subtilisin kexin 9 (PCSK9) gene in cholesterol metabolism and disease. Hum Mutat. 2009;30:520–9. 10.1002/humu.20882. 19191301 10.1002/humu.20882

[52] Cohen JC, Boerwinkle E, Mosley TH Jr., Hobbs HH. Sequence variations in PCSK9, low LDL, and protection against coronary heart disease. N Engl J Med. 2006;354:1264–72. 10.1056/NEJMoa054013. 16554528 10.1056/NEJMoa054013

[53] Larrea-Sebal A, Jebari-Benslaiman S, Galicia-Garcia U, Jose-Urteaga AS, Uribe KB, Benito-Vicente A, et al. Predictive Modeling and Structure Analysis of Genetic Variants in Familial Hypercholesterolemia: Implications for Diagnosis and Protein Interaction Studies. Curr Atheroscler Rep Springer. 2023;25:839–59. 10.1007/S11883-023-01154-7/FIGURES/5. 10.1007/s11883-023-01154-7 PMC10618353 37847331

[54] Khalil YA, Rabès JP, Boileau C, Varret M. APOE gene variants in primary dyslipidemia. Atherosclerosis Elsevier. 2021;328:11–22. 10.1016/J.ATHEROSCLEROSIS.2021.05.007. 10.1016/j.atherosclerosis.2021.05.007 34058468

[55] Dastani Z, Hivert MF, Timpson N, Perry JR, Yuan X, Scott RA, et al. Novel loci for adiponectin levels and their influence on type 2 diabetes and metabolic traits: a multi-ethnic meta-analysis of 45,891 individuals. PLoS Genet. 2012;8:e1002607. . 10.1371/journal.pgen.1002607PGENETICS-D-11-02099 22479202 10.1371/journal.pgen.1002607 PMC3315470

[56] Civeira F, Martín C, Cenarro A. APOE and familial hypercholesterolemia. Curr Opin Lipidol Curr Opin Lipidol. 2024;35:195–9. 10.1097/MOL.0000000000000937. 38640077 10.1097/MOL.0000000000000937

[57] Cenarro A, Etxebarria A, de Castro-Orós I, Stef M, Bea AM, Palacios L, et al. The p.Leu167del Mutation in APOE Gene Causes Autosomal Dominant Hypercholesterolemia by Down-regulation of LDL Receptor Expression in Hepatocytes. J Clin Endocrinol Metab. 2016;101:2113–21. 10.1210/jc.2015-3874. 27014949 10.1210/jc.2015-3874

[58] Bello-Álvarez D, Cenarro A, Bea AM, Marco-Benedí V, Jarauta E, Ortiz-Palma P, et al. APOE p.(Leu167del) variant in hypercholesterolemia: Prevalence & phenotypic expression. J Clin Lipidol Elsevier BV. 2025. 10.1016/j.jacl.2025.09.016. 10.1016/j.jacl.2025.09.016 41198423

[59] D’Erasmo L, Minicocci I, Nicolucci A, Pintus P, Van Roeters JE, Masana L, et al. Autosomal Recessive Hypercholesterolemia: Long-Term Cardiovascular Outcomes. J Am Coll Cardiol. 2018;71:279–88. 10.1016/j.jacc.2017.11.028. 29348020 10.1016/j.jacc.2017.11.028

[60] Cuchel M, Raal FJ, Hegele RA, Al-Rasadi K, Arca M, Averna M, et al. 2023 Update on European Atherosclerosis Society Consensus Statement on Homozygous Familial Hypercholesterolaemia: new treatments and clinical guidance. Eur Heart J Eur Heart J. 2023;44:2277–91. 10.1093/EURHEARTJ/EHAD197. 37130090 10.1093/eurheartj/ehad197 PMC10314327

[61] Sánchez-Hernández RM, Ibarretxe D, Fuentes Jiménez F, Martínez-Hervás S, Blanco-Echevarría A, Cortés Rodríguez B, et al. Homozygous Familial Hypercholesterolemia in Spain: Data From Registry of the Spanish Atherosclerosis Society. J Clin Endocrinol Metab J Clin Endocrinol Metab. 2025;110:2280–7. 10.1210/CLINEM/DGAE784. 39514762 10.1210/clinem/dgae784

[62] Reijman MD, Tromp TR, Hutten BA, Hovingh GK, Blom DJ, Catapano AL, et al. Cardiovascular outcomes in patients with homozygous familial hypercholesterolaemia on lipoprotein apheresis initiated during childhood: long-term follow-up of an international cohort from two registries. Lancet Child Adolesc Heal Elsevier B V. 2024;8:491–9. 10.1016/S2352-4642(24)00073-7. 10.1016/S2352-4642(24)00073-7 PMC11963317 38759658

[63] Maurer ME, Cooper JA. The adaptor protein Dab2 sorts LDL receptors into coated pits independently of AP-2 and ARH. J Cell Sci J Cell Sci. 2006;119:4235–46. 10.1242/JCS.03217. 16984970 10.1242/jcs.03217

[64] Filigheddu F, Quagliarini F, Campagna F, Secci T, Degortes S, Zaninello R, et al. Prevalence and clinical features of heterozygous carriers of autosomal recessive hypercholesterolemia in Sardinia. Atherosclerosis Atherosclerosis. 2009;207:162–7. 10.1016/J.ATHEROSCLEROSIS.2009.04.027. 19477448 10.1016/j.atherosclerosis.2009.04.027

[65] Arca M, Zuliani G, Wilund K, Campagna F, Fellin R, Bertolini S, et al. Autosomal recessive hypercholesterolaemia in Sardinia, Italy, and mutations in ARH: A clinical and molecular genetic analysis. Lancet Elsevier B V. 2002;359:841–7. 10.1016/S0140-6736(02)07955-2. 10.1016/S0140-6736(02)07955-2 11897284

[66] Rios J, Stein E, Shendure J, Hobbs HH, Cohen JC. Identification by whole-genome resequencing of gene defect responsible for severe hypercholesterolemia. Hum Mol Genet. 2010;19:4313–8. 10.1093/hmg/ddq352. 20719861 10.1093/hmg/ddq352 PMC2957323

[67] Patel SB. Recent advances in understanding the STSL locus and ABCG5/ABCG8 biology. Curr Opin Lipidol. 2014;25:169–75. 10.1097/MOL.0000000000000071. 24811295 10.1097/MOL.0000000000000071

[68] Nomura A, Emdin CA, Won HH, Peloso GM, Natarajan P, Ardissino D, et al Heterozygous ATP-binding cassette transporter g5 gene deficiency and risk of coronary artery disease. Circ Genomic Precis Med. 2020;13:417. 10.1161/CIRCGEN.119.002871 10.1161/CIRCGEN.119.002871 PMC7983048 32862661

[69] Tada H, Nomura A, Yamagishi M, Kawashiri M, aki. First case of sitosterolemia caused by double heterozygous mutations in ABCG5 and ABCG8 genes. J Clin Lipidol Elsevier Ltd. 2018;12:1164–e11684. 10.1016/j.jacl.2018.06.003. 10.1016/j.jacl.2018.06.003 30007774

[70] Alves AC, Chora JR, Miranda B, Medeiros AM, Graça R, Bañares VG, et al. Sitosterolemia in Iberoamerican countries: 16 new cases and phenotype genotype analysis. J Clin Lipidol Elsevier. 2025;19:1715–24. 10.1016/J.JACL.2025.08.020. 10.1016/j.jacl.2025.08.020 41130816

[71] Tada MT, Rocha VZ, Lima IR, Oliveira TGM, Chacra AP, Miname MH, et al. Screening of ABCG5 and ABCG8 Genes for Sitosterolemia in a Familial Hypercholesterolemia Cascade Screening Program. Circ Genomic Precis Med. Circ Genom Precis Med. 2022;15:E003390. 10.1161/CIRCGEN.121.003390. 35549507 10.1161/CIRCGEN.121.003390

[72] Dron JS, Hegele RA. Genetics of Lipid and Lipoprotein Disorders and Traits. Curr Genet Med Rep. Curr Genet Med Rep. 2016;4:130–41. 10.1007/S40142-016-0097-Y. 28286704 10.1007/s40142-016-0097-y PMC5325854

[73] Reeskamp LF, Volta A, Zuurbier L, Defesche JC, Hovingh GK, Grefhorst A. ABCG5 and ABCG8 genetic variants in familial hypercholesterolemia. J Clin Lipidol Elsevier. 2020;14(7):207–e217. 10.1016/J.JACL.2020.01.007. 10.1016/j.jacl.2020.01.007 32088153

[74] Lee M-H, Lu K, Hazard S, Yu H, Shulenin S, Hidaka H, et al. Identification of a gene, ABCG5, important in the regulation of dietary cholesterol absorption. Nat Genet. 2001;27:79–83. 10.1038/83799 11138003 10.1038/83799 PMC1350991

[75] Yoo E-G. Sitosterolemia: a review and update of pathophysiology, clinical spectrum, diagnosis, and management. Ann Pediatr Endocrinol Metab. 2016;21:7–14. 10.6065/apem.2016.21.1.7. 27104173 10.6065/apem.2016.21.1.7 PMC4835564

[76] Williams K, Segard A, Graf GA, Sitosterolemia. Twenty Years of Discovery of the Function of ABCG5ABCG8. Int J Mol Sci Int J Mol Sci. 2021;22:1–14. 10.3390/IJMS22052641. 10.3390/ijms22052641 PMC7961684 33807969

[77] Rees DC, Iolascon A, Carella M, O’Marcaigh AS, Kendra JR, Jowitt SN, et al. Stomatocytic haemolysis and macrothrombocytopenia (Mediterranean stomatocytosis/macrothrombocytopenia) is the haematological presentation of phytosterolaemia. Br J Haematol Br J Haematol. 2005;130:297–309. 10.1111/J.1365-2141.2005.05599.X. 16029460 10.1111/j.1365-2141.2005.05599.x

[78] Korbelius M, Kuentzel KB, Bradić I, Vujić N, Kratky D. Recent insights into lysosomal acid lipase deficiency. Trends Mol Med. 2023;29:425. 10.1016/J.MOLMED.2023.03.001. 37028992 10.1016/j.molmed.2023.03.001 PMC7614602

[79] Pullinger CR, Stock EO, Movsesyan I, Malloy MJ, Frost PH, Tripuraneni R, et al. Identification and metabolic profiling of patients with lysosomal acid lipase deficiency. J Clin Lipidol. 2015;9(1):716–e726. 10.1016/j.jacl.2015.07.008. 26350820 10.1016/j.jacl.2015.07.008

[80] Chora JR, Alves AC, Medeiros AM, Mariano C, Lobarinhas G, Guerra A, et al. Lysosomal acid lipase deficiency: A hidden disease among cohorts of familial hypercholesterolemia? J Clin Lipidol. 2017;11:477–e4842. 10.1016/j.jacl.2016.11.002. 28502505 10.1016/j.jacl.2016.11.002

[81] Sustar U, Groselj U, Trebusak Podkrajsek K, Mlinaric M, Kovac J, Thaler M, et al. Early Discovery of Children With Lysosomal Acid Lipase Deficiency With the Universal Familial Hypercholesterolemia Screening Program. Front Genet Front Media S A. 2022;13:936121. 10.3389/FGENE.2022.936121/BIBTEX. 10.3389/fgene.2022.936121 PMC9314654 35903350

[82] Fornengo P, Ferro A, Fagoonee S, Rinaudo E, Amione C, Durazzo M. Lysosomal acid lipase deficiency: the forgotten link between liver and cardiovascular disease. World J Cardiol World J Cardiol. 2025;17. 10.4330/WJC.V17.I11.111292. 10.4330/wjc.v17.i11.111292 PMC12678881 41356588

[83] Vijay S, Evans J, Lacaille F, Abel F, Heras J. de las. Survival, growth, and safety findings in patients with rapidly progressive, infantile-onset LAL-D: Results from the international LAL-D registry. Mol Genet Metab. 2025;146:109290. 10.1016/j.ymgme.2025.109290 10.1016/j.ymgme.2025.109290 41270440

[84] Jones SA, Rojas-Caro S, Quinn AG, Friedman M, Marulkar S, Ezgu F, et al. Survival in infants treated with sebelipase Alfa for lysosomal acid lipase deficiency: an open-label, multicenter, dose-escalation study. Orphanet J Rare Dis. 2017;12:25. 10.1186/S13023-017-0587-3. 28179030 10.1186/s13023-017-0587-3 PMC5299659

[85] Vijay S, Brassier A, Ghosh A, Fecarotta S, Abel F, Marulkar S, et al. Long-term survival with sebelipase alfa enzyme replacement therapy in infants with rapidly progressive lysosomal acid lipase deficiency: final results from 2 open-label studies. Orphanet J Rare Dis. 2021;16:13. 10.1186/S13023-020-01577-4. 33407676 10.1186/s13023-020-01577-4 PMC7789691

