# Supplementary material for: The Spectrum of Genetic Causes of Familial Hypercholesterolemia Phenotype
Source: Curr Atheroscler Rep. 2026 Jul 4;28(1):67. doi: 10.1007/s11883-026-01435-x (PMC13332989; doi:10.1007/s11883-026-01435-x)
Supplement: Supplementary file 1 — Supplementary Material 1 [file 11883_2026_1435_MOESM1_ESM.docx]

Supplementary Table 1. *LDLR* variants submitted to ClinVar by variant type and classification.

| **Variant type** | **Pathogenic/ Likely pathogenic** | **Benign/Likely benign** | **Uncertain significance** | **Conflicting classifications of pathogenicity** | **classification not provided** | **Total** |
| --- | --- | --- | --- | --- | --- | --- |
| missense | 726 | 41 | 775 | 210 | 8 | 1,760 |
| frameshift | 600 | 1 | 6 | 2 | 1 | 610 |
| nonsense | 237 |  |  |  | 1 | 238 |
| CNV - deletion | 260 |  | 16 | 8 | 3 | 287 |
| CNV - duplication | 69 |  | 17 | 7 | 1 | 94 |
| in frame indel | 89 |  | 39 | 14 | 1 | 143 |
| splicing | 200 | 280 | 68 | 32 | 6 | 586 |
| synonymous | 3 | 531 | 19 | 26 |  | 579 |
| intronic | 2 | 80 | 12 | 2 | 11 | 107 |
| cis variant | 7 |  |  |  |  | 7 |
| 3'UTR |  | 49 | 72 | 2 | 5 | 128 |
| 5'UTR | 15 | 9 | 104 | 10 | 1 | 139 |
| **Total** | **2,208** | **991** | **1,128** | **313** | **38** | **4,678** |
