# Supplementary material for: The Spectrum of Genetic Causes of Familial Hypercholesterolemia Phenotype
Source: Curr Atheroscler Rep. 2026 Jul 4;28(1):67. doi: 10.1007/s11883-026-01435-x (PMC13332989; doi:10.1007/s11883-026-01435-x)
Supplement: Supplementary file 2 — Supplementary Material 2 [file 11883_2026_1435_MOESM2_ESM.docx]

Supplementary Table 2. *APOB* variants submitted to ClinVar by variant type and classification.

| **Variant type** | **Pathogenic/ Likely pathogenic** | **Benign/Likely benign** | **Uncertain significance** | **Conflicting classifications of pathogenicity** | **classification not provided** | **Total** |
| --- | --- | --- | --- | --- | --- | --- |
| missense | 15 | 345 | 1,707 | 652 | 5 | 2,724 |
| frameshift | 107 | 1 | 11 | 2 |  | 121 |
| nonsense | 79 |  | 16 | 3 | 1 | 99 |
| CNV - deletion | 4 |  |  |  |  | 4 |
| CNV - duplication |  |  | 1 |  |  | 1 |
| in frame indel |  | 4 | 23 | 3 |  | 30 |
| splicing | 33 | 177 | 32 | 23 | 1 | 266 |
| synonymous |  | 1,305 | 19 | 87 |  | 1,411 |
| intronic |  | 8 | 2 |  |  | 10 |
| 3'UTR |  |  | 6 | 5 |  | 11 |
| 5'UTR |  | 5 | 5 | 2 |  | 12 |
| **Total** | **238** | **1,845** | **1,822** | **777** | **7** | **4,689** |
