# Supplementary material for: The Spectrum of Genetic Causes of Familial Hypercholesterolemia Phenotype
Source: Curr Atheroscler Rep. 2026 Jul 4;28(1):67. doi: 10.1007/s11883-026-01435-x (PMC13332989; doi:10.1007/s11883-026-01435-x)
Supplement: Supplementary file 3 — Supplementary Material 3 [file 11883_2026_1435_MOESM3_ESM.docx]

Supplementary Table 3. *PCSK9* variants submitted to ClinVar by variant type and classification.

| **Variant type** | **Pathogenic/Likely pathogenic** | **Benign/Likely benign** | **Uncertain significance** | **Conflicting classifications of pathogenicity** | **classification not provided** | **Total** |
| --- | --- | --- | --- | --- | --- | --- |
| missense | 16 | 26 | 556 | 86 |  | 684 |
| frameshift | 1 | 29 | 12 | 3 | 1 | 46 |
| nonsense |  | 15 | 6 | 5 |  | 26 |
| CNV - deletion |  |  | 2 |  |  | 2 |
| CNV - duplication |  |  | 1 |  |  | 1 |
| in frame indel |  | 5 | 15 | 6 |  | 26 |
| splicing | 1 | 142 | 46 | 18 | 3 | 210 |
| synonymous |  | 283 | 13 | 20 |  | 316 |
| intronic |  | 34 | 17 |  | 1 | 52 |
| 3'UTR |  | 15 | 33 | 4 |  | 52 |
| 5'UTR |  | 6 | 18 | 4 |  | 28 |
| **Total** | **18** | **555** | **719** | **146** | **5** | **1,443** |
